# Repulsive Guidance Molecule b Deficiency Induces Gut Microbiota Dysbiosis and Increases the Susceptibility to Intestinal Inflammation in Mice

**DOI:** 10.3389/fmicb.2021.648915

**Published:** 2021-04-28

**Authors:** Ying Shi, Lu Zhong, Yuting Li, Yanfang Chen, Shufen Feng, Min Wang, Yin Xia, Shaohui Tang

**Affiliations:** ^1^Department of Gastroenterology, The First Affiliated Hospital, Jinan University, Guangzhou, China; ^2^School of Biomedical Sciences, Faculty of Medicine, The Chinese University of Hong Kong, Shatin, Hong Kong; ^3^Department of Gastroenterology, Hepatology and Infectious Diseases, University Hospital Tübingen, Tübingen, Germany

**Keywords:** Rgmb deficiency, colitis, gut microbiota, *Prevotellaceae*, intestinal mucosa

## Abstract

Imbalance of gut microbiota can induce or aggravate intestinal inflammation. To enhance our understanding of the molecular mechanisms of gut microbiota and inflammatory bowel disease (IBD), we studied the role of repulsive guidance molecule b (RGMb) in gut microbiota and colitis in mice. We generated Rgmb knockout mice and inducible Rgmb knockout mice and induced colitis using dextran sulfate sodium (DSS) in these mice. 16S ribosomal RNA (rRNA) high-throughput sequencing was performed to acquire the gut microbiota composition and abundance. We found that Rgmb deficiency significantly altered the diversity of gut microbiota and also induced dysbiosis. In sharp contrast to the balanced distribution of various bacteria in control mice, *Prevotellaceae* was almost exhausted in Rgmb-deficient mice under both basal and inflammatory conditions. Correlation analysis indicated that *Prevotellaceae* was negatively associated with inflammation in Rgmb-deficient mice with colitis. Similar results were obtained at the early inflammatory stage of colitis associated colon cancer (CAC). Taken together, our results reveal that Rgmb deficiency leads to dysbiosis of predominant gut microbiota under basal and inflammatory conditions. Rgmb-deficiency-mediated *Prevotellaceae* loss may render mice more susceptible to intestinal inflammation. Therefore, RGMb may be a novel potential target for reconstruction of the gut microbiota for the treatment of IBD.

## Introduction

Inflammatory bowel disease (IBD) including ulcerative colitis (UC) and Crohn’s disease (CD) can be induced by multiple causes (Feuerstein et al., 2020; Torres et al., 2020). In addition to the environmental, genetic, and immune factors, dysbiosis of microbiota has been found to contribute to the initiation and development of IBD (Aschard et al., 2019; Lloyd-Price et al., 2019; Ansari et al., 2020). Accumulating evidence demonstrates that IBD is associated with altered gut microbiome and immune abnormalities (Chen et al., 2014; Imhann et al., 2018; Franzosa et al., 2019). Rebuilding the normal gut microbiota has become a new strategy for the IBD treatment. Specific genetic deficiency weakens the mucosal defense ability and increases the susceptibility to colitis. Although the important role of gut microbiota has been widely accepted, the mechanisms of dysbiosis are not fully understood. Exploring the genetic contributions to gut microbiota contributes to our understanding of the mechanisms underlying IBD development (Aschard et al., 2019).

Intestinal chronic inflammation is an established risk factor for colorectal cancer (CRC) in patients with IBDs. With the colitis progression and the mucosal epithelial barrier injury, the risk of CRC increases (Dupaul-Chicoine et al., 2010). Inflammatory epithelial cells produce reactive oxygen species, which affect carcinogenesis-related genes and proteins via genetic or epigenetic modifications (Chiba et al., 2012; Aviello et al., 2019). The intestinal epithelium is exposed to a huge burden of foreign antigens and various microorganisms, which collectively contribute to the development of intestinal inflammation into carcinoma (Plichta et al., 2019).

Repulsive guidance molecule b (RGMb/Dragon) is a member of RGM family, which consists of RGMa, RGMb, and RGMc. RGMb is a co-receptor for bone morphogenetic proteins (BMPs) (Xia et al., 2007). Previously, we found that RGMb was highly expressed in macrophages, and RGMb is an important negative regulator of IL-6 expression in immune cells (Xia et al., 2011). RGMb promoted CRC via the Erk1/2-BMP4-Smad1/5/8 pathways and induced oxaliplatin resistance by inhibiting JNK and p38 MAPK activation (Shi et al., 2015, 2016).

RGMb is highly expressed in normal resting lung interstitial macrophages and alveolar epithelial cells, whereas programmed death ligand-2 (PD-L2) is expressed in lung dendritic cells. It has been reported that PD-L2 and BMP-2/4 bind to distinct sites on RGMb (Xiao et al., 2014). Blockade of the interaction between RGMb and PD-L2 markedly interfered with the initial T cell expansion required for respiratory tolerance and impaired the development of respiratory tolerance. PD-1 inhibits antitumor immunity, while RGMb regulates respiratory immunity (Xiao et al., 2014; Ohaegbulam et al., 2015). PD-L2 binds to both PD-1 and RGMb. These observations suggest that RGMb may play an important role in immune and inflammatory disorders. In the present study, we examined the gut microbiota in Rgmb-deficient mice under basal conditions, and after induction of colitis, we found dysbiosis of gut microbiota in those mice. Our results suggest that RGMb plays an important role in controlling microbiota homeostasis.

## Materials and Methods

### Ethics Statement

All procedures involving experimental animals were performed in accordance with protocols that were approved by the Ethics Committee for Animal Research of Jinan University, Guangzhou, China (No. IACUC-20180316-01) and complied with the Guide for the Care and Use of Laboratory Animals (NIH publication No. 86-23, revised in 1985).

### Global Rgmb Knockout Mice

Rgmb^*flox/flox*^ mice on C57BL/6 background have been described (Liu et al., 2018). Stra8-icre mice were purchased from Jackson Laboratory (Stock No: 017490). In the Stra8-icre transgenic line, cre is expressed in pre-meiotic germ cells. To obtain global Rgmb knockout (gKO) mice, Rgmb^*flox/flox*^ mice were mated with Stra8-icre mice to Rgmb^*flox/wt*^; Stra8-icre mice, which then were intercrossed to obtain heterozygous gKO. Heterozygous animals were bred to obtain heterozygous and homozygous Rgmb-null (Rgmb^*gKO*^) mice. Mice were housed under specific pathogen-free (SPF) conditions with a light/dark cycle of 12/12 h.

### Inducible Rgmb Knockout Mice

Inducible knockout mice (igKO) were obtained by crossbreeding of Rgmb^*flox/flox*^ with ROSA26-CreERT2 mice on the C57BL/6 background. Customized food pellets with Tamoxifen (Sigma–Aldrich, T5648), with a concentration of 500 mg Tamoxifen/1 kg pellets, were provided to induce global deletion of the Rgmb gene.

### DSS-Induced Colitis in Mice

The inducible Rgmb knockout and control mice were free to drink fresh 3% (w/v) dextran sulfate sodium (DSS) (31404, Sigma) solution continuously for 7 days to induce colitis. Control mice were free to drink distilled water only. Body weights and disease activity index (DAI) were monitored. Serum and colon tissues were harvested for analysis.

### AOM/DSS-Induced Colitis Associated Colon Cancer in Mice

Mice were injected intraperitoneally with AOM at a dose of 12.5 mg/kg body weight. One week after the injection, 3% DSS was administered to mice via their drinking water for 7 days, and they then were switched to normal drinking water for 16 days. The treatments were repeated for three cycles.

### Immunohistochemical Staining

Colon tissue samples were formalin-fixed, paraffin-embedded, and sectioned. Antigen was retrieved by Citrate Antigen Retrieval solution (Maxim, Fuzhou, China). Sections were treated with peroxidase and blocked with 10% donkey serum. The sections were incubated with the RGMb antibody (AF3630, R&D Systems, Minneapolis, MN, United States) overnight at 4°C. After washes, the sections were incubated with anti-goat secondary antibodies (HAF109, R&D Systems, Minneapolis, MN, United States) and DAB Detection Kit (Maxim, Fuzhou, China) before they were counterstained with hematoxylin.

### ELISA

Serum was collected from DSS-induced colitis mice and measured using the IL-10 (EMC005.48), IL-6 (EMC004.48), TNF-α (EMC102a.48), and INF-γ (EMC101g.48) ELISA kits (Neobioscience, China) according to the manufacturer’s instructions. Detection reagents A and B were added following the substrate solution, and the reaction was terminated with the STOP solution. The absorbance at 450 nm was measured using a microplate reader.

### Fecal Sample Collection and DNA Extraction

Fresh feces were collected from heterozygous Rgmb^*gKO*^ (Rgmb^±^) and wildtype (Rgmb^+/+^) mice at 10 weeks of age, and from Rgmb^*igKO*^ and control mice after the end of Tamoxifen administration. Each fecal sample was pooled from five littermates. DNAs of fecal samples were extracted using QIAamp Fast DNA Stool Mini Kit (Qiagen, DE). Quality of the extracted DNAs was evaluated by 1.5% agarose gel electrophoresis in Tris-Acetate-EDTA buffer. DNA samples were stored at −20°C until library preparation and 16S ribosomal RNA (rRNA) sequencing.

### Library Preparation and 16S rRNA Sequencing

The V4 hypervariable region of the bacterial 16S rRNA was amplified with the forward primer 515F (5′-GTGYCAGCMGCCGCGGTAA-3′) and the reverse primer 806R (5′-GGACTACNVGGGTWTCTAAT-3′) using KAPA HiFi HotStart ReadyMix (KAPA Biosystems, United States). The PCR products were purified using an AxyPrep PCR Cleanup Kit (Axygen, United States) following the manufacturer’s protocol. Paired-end sequencing was performed using Illumina FC-420-1004 MiniSeq Mid Output Kit (Illumina, United States) based on an Illumina MiniSeq System (Illumina, United States).

The raw paired-end reads were assembled and merged by FLASH (Magoc and Salzberg, 2011; Zhang et al., 2019). The PCR primers were subsequently truncated by cutadapt (Martin, 2011). The quality-controlled sequences were further chimerically removed and OUT-clustered by Usearch (Edgar, 2013). In detail, all reads were demultiplexed into one file, clustered at 97% similarity, and then the chimera checking was performed using UCHIME in reference mode. Representative sequences were generated, singletons were removed, and then a final operational taxonomic unit (OTU) table was created. The representative sequences of OTU were aligned on the Silva database for taxonomic classification by RDP Classifier.

### Bioinformatics and Statistical Analyses

Alpha-diversity (α-diversity) metrics [ACE, Shannon and Pielou’s evenness index (J)] and beta-diversity (β-diversity) metrics [principal component analysis (PCA)] were all performed by R package vegan (version 2.5-5) (Oksanen et al., 2015). The taxa abundance in different levels was measured and plotted using ggplot2 (Wickham, 2009). LEfSe analysis was performed to identify taxa with differentiating abundance in the different group with LEfSe1.0 (Segata et al., 2011). The differential bacteria taxa in different groups were screened by wilcox.test with the *P*-value adjustment method FDR. ANOVA tests were either conducted by a one-way test or pairwise *t*-test with the *P*-value adjusted to the “BH” method for multiple comparisons or the Bartlett’s test using the equal variance and Duncan’s method (package laercio) to group differences. Pearson’s correlation coefficient was statistically analyzed by IBM SPSS statistics 25.0.

Statistical analysis for non-genomics data was performed using SPSS 21.0 software (SPSS Inc., Chicago, IL, United States). Data between two groups were compared by using Student’s *t*-test (means ± standard deviation). Pearson’s correlation coefficient (*r*) is used to analyze the correlation between two variables. All of the values are expressed as the mean ± SD of at least three independent experiments performed in triplicate, and *P* < 0.05 was considered to be statistically significant. Graphs were plotted using GraphPad Prism 8 software (GraphPad Software Inc., San Diego, CA, United States).

### Data Availability

The 16S sequencing raw reads of this research are available on the NCBI SRA. The accession number for the 16S rRNA sequencing data reported in this paper is NCBI SRA: PRJNA690936.

## Results

### Rgmb-Deficiency Exacerbated Colitis in Mice

**FIGURE 1:**
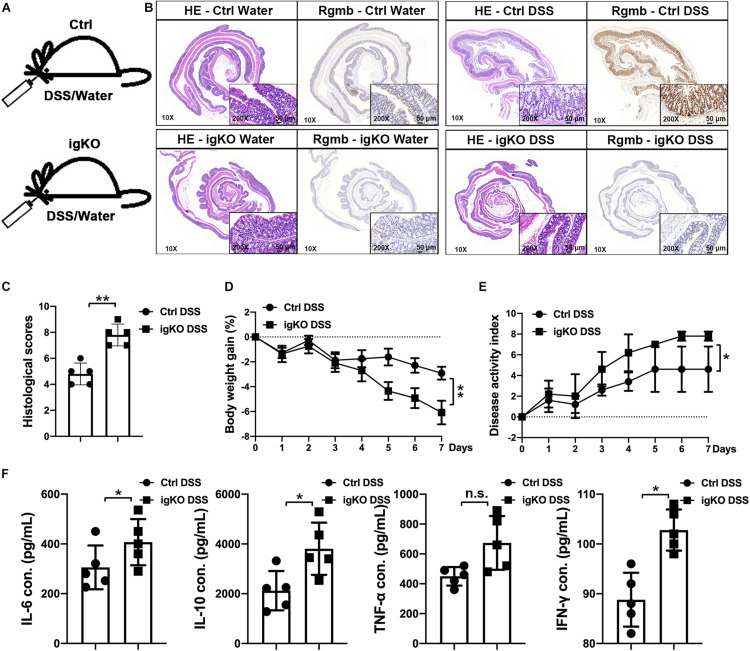
More severe colitis in Rgmb-deficient mice. **(A)** The schematic diagram showing the protocol for induction of colitis by DSS in Rgmb-deficient (igKO) and control (Ctrl) mice. **(B)** H&E and IHC staining. H&E staining shows colon tissues of normal or colitis mice with or without DSS treatment. IHC staining indicates that Rgmb was expressed in the colons of control mice, but the signal was lost in Rgmb igKO colons. Brown color represents staining for RGMb protein. Blue represents the nuclei. **(C)** Histological scores of intestinal tissues of Rgmb-deficient and control mice with colitis. **(D)** Body weights of colitic Rgmb igKO and control mice. **(E)** DAI of colitic Rgmb igKO and control mice. **(F)** Serum concentrations of serum IL-6, IL-10, TNF-α, and INF-γ in colitic Rgmb igKO and control mice. *n* = 5, *P < 0.05, **P < 0.01, and n.s., not significant.

In order to explore the role of Rgmb in colitis, we induced colitis in Rgmb-deficient (Rgmb^*igKO*^, Supplementary Figure 1a) and control mice using 3% DSS in drinking water for 7 consecutive days (Figure 1A). Immunohistochemistry showed that naive control mice exhibited RGMb expression in both epithelial and parenchymal cells, while this expression was absent in Rgmb-deficient mice. Consistent with our previous observation (Shi et al., 2015), RGMb expression in the colonic epithelial cells was increased after DSS treatment in control mice (Figure 1B). Histological examination demonstrated a loss of crypts, infiltration of inflammatory cells into the mucosa and submucosa, and edema of the submucosa in control mice, and these phenotypes were much more severe in Rgmb-deficient mice. Mucosal injury scores were dramatically increased in Rgmb-deficient mice compared with control mice (Figure 1C). Body weights were much more decreased in Rgmb-deficient mice than in control mice as the colitis progressed (Figure 1D). DAI of Rgmb-deficient mice was dramatically increased compared with control mice (Figure 1E). After 7 days of DSS administration, colitis was observed in both Rgmb-deficient and control mice. Serum concentrations of IL-6, IL-10, TNF-α (*P* = 0.05), and INF-γ were higher in Rgmb-deficient mice than in control mice (Figure 1F). Thus, deletion of Rgmb exacerbated colitis.

### Rgmb Deficiency Induced Dysbiosis of Gut Microbiota in Mice With Colitis

**FIGURE 2:**
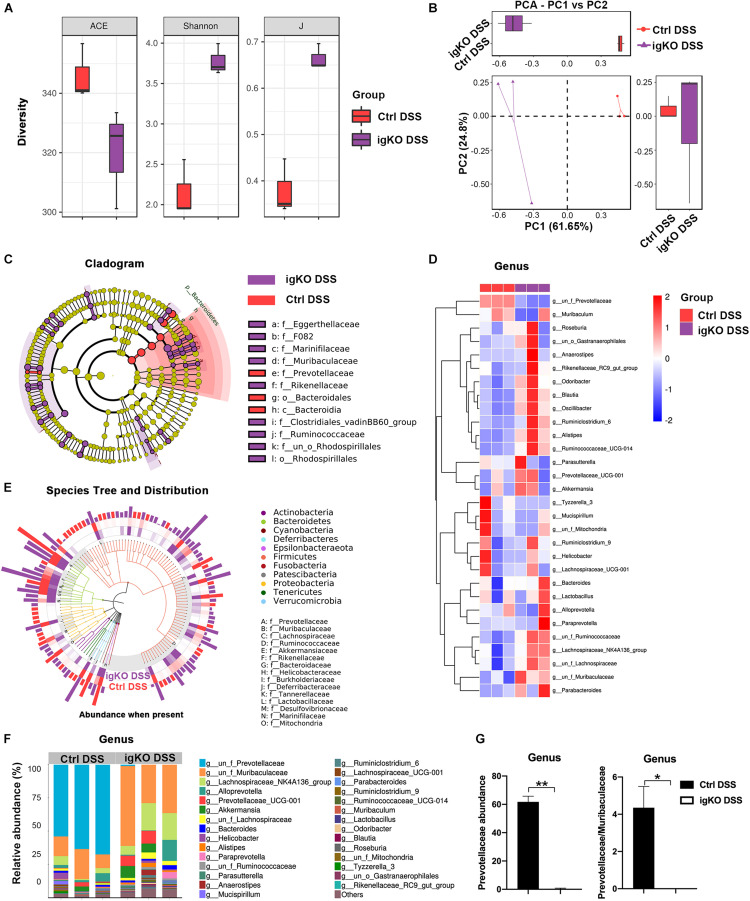
Dysbiosis of gut microbiota in colitic Rgmb-deficient mice. **(A)** Rgmb-deficiency altered α-diversity of gut microbiota in mice with colitis. **(B)** OTUs indicate significant compositional discriminations of fecal microbiota by PCA between Rgmb-deficient and control colitis mice. **(C)** The cladogram of evolution relationship between gut microbiota from Rgmb-deficient and control mice with colitis. **(D)** Heatmap represents the relative abundance of gut microbiota from Rgmb-deficient and control mice with colitis at genus level. **(E)** The species tree and distribution of gut bacterium at species level in Rgmb-deficient and control mice with colitis. **(F)** The relative abundance of gut microbiota in Rgmb-deficient and control mice with colitis at genus level. **(G)** The relative abundance of *Prevotellaceae* (left panel) and the ratio of *Prevotellaceae* to *Muribaculaceae* (right panel) in Rgmb-deficient and corresponding control mice with colitis at genus level. *n* = 3. Each fresh fecal sample was pooled from five littermate mice. ^∗^*P* < 0.05 and ^∗∗^*P* < 0.01.

To understand the mechanisms by which Rgmb deficiency promoted colitis, we pooled fresh feces from littermates of Rgmb-deficient and control mice after colitis induction, and performed 16s rRNA gene sequencing for fecal microbiota. As shown by ACE index, reduced species and community richness were observed in Rgmb-deficient mice (Figure 2A). Shannon indexes indicated that more gut microbial diversities were captured with the current sequencing depth in Rgmb-deficient mice than in control mice. J index demonstrated a significant rise in species evenness in Rgmb-deficient mice (Figure 2A). Observed OTUs from weighted Unifrac PCA showed significant compositional discriminations of fecal microbiota between Rgmb-deficient and control mice (Figure 2B).

Colitis associated bacterial taxa were determined by differential abundance analysis. Several bacterial OTUs were differentially abundant in Rgmb-deficient mice compared with control mice in different taxonomic ranks (Figures 2C,D). Moreover, bacterial richness was correspondingly altered by Rgmb-deficiency in colitic mice. We observed enrichments of *Muribaculaceae*, *Lachnospiraceae*, *Ruminococcaceae*, *Akkermansiaceae*, *Rikenellaceae*, *Bacteroidaceae*, *Burkholderiaceae*, *Tannerellaceae*, *Lactobacillaceae*, and *Marinifilaceae*, and reduction of *Prevotellaceae*, *Helicobacteraceae*, *Deferribacteraceae*, *Desulfovibrionaceae*, and *Mitochondria* in Rgmb-deficient colitis mice as shown by genus taxonomic rank (Figure 2E). Of note, depletion of *Prevotellaceae* was observed in Rgmb-deficient mice (Figures 2F,G, left panel). We also analyzed other bacteria and found that significant differences were only observed in *Ruminococcaceae* and *Odoribacter* (Supplementary Figure 2). Although there was no significant difference in *Muribaculaceae*, but the ratio of *Prevotellaceae* to *Muribaculaceae* was significantly reduced in Rgmb-deficient mice compared with control mice (Figure 2G, right panel). These results indicate that Rgmb deficiency leads to gut microbiota dysbiosis in DSS-induced colitis.

We also collected fresh feces from Rgmb-deficient or control mice under basal conditions for 16s rRNA gene sequencing. OTUs were differentially abundant in Rgmb-deficient mice compared with control mice in different taxonomic ranks, and bacterial richness was correspondingly changed (Supplementary Figures 1b,c). We also found enrichments of *Muribaculaceae*, *Ruminococcaceae*, *Akkermansiaceae*, *Bacteroidaceae*, *Helicobacteraceae*, *Desulfovibrionaceae*, *Tannerellaceae*, and *Marinifilaceae*, and reduction of *Prevotellaceae*, *Lachnospiraceae*, *Burkholderiaceae*, *Rikenellaceae*, *Erysipelotrichaceae*, *Lactobacillaceae*, and *Mitochondria* in Rgmb-deficient mice (Supplementary Figure 1d). Strikingly, depletion of *Prevotellaceae* and decreased ratio of *Prevotellaceae* to *Muribaculaceae* were observed in Rgmb-deficient mice even under basal conditions (Supplementary Figures 1e,f). These results indicate that Rgmb deficiency induces dysbiosis of gut microbiota in both normal and colitic mice.

### *Prevotellaceae* Was Negatively Correlated With Inflammation in Rgmb-Deficient Mice With Colitis

**FIGURE 3:**
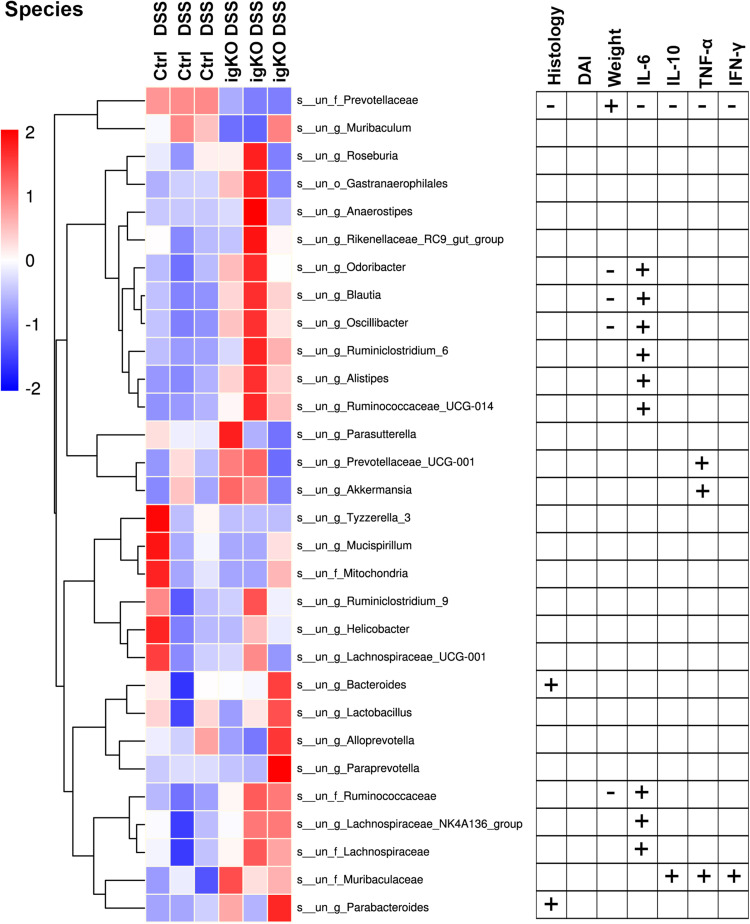
Correlation analysis of bacterium abundance and colitis-associated characteristics. The heatmap of the alterations in gut microbiota between Rgmb-deficient and control mice with colitis (left panel). Statistical analyses on the correlations between differential bacteria and inflammation in Rgmb-deficient mice with colitis (right panel). Each fresh fecal sample was pooled from five littermate mice. + Positive correlation with statistical significance. - Negative correlation with statistical significance.

Since *Prevotellaceae* depletion was observed in Rgmb-deficient mice, we hypothesize that *Prevotellaceae* plays a role in colitis in Rgmb-deficient mice. We analyzed the correlation of *Prevotellaceae* abundance with colitis associated characteristics (Figure 3). The abundance of *Prevotellaceae* was negatively correlated with histological mucosal injury and inflammatory cytokines including IL-6, IL-10, TNF-α, and INF-γ, whereas positively associated with body weights.

Correlation analyses were also performed between other fecal bacteria and inflammation in Rgmb-deficient mice with colitis (Figure 3 and Supplementary Table 1). We found that other bacteria were corrected with colitis-associated characteristics to various degrees. *Muribaculaceae* was positively correlated with serum IL-10, TNF-α, and INF-γ. Changes in *Bacteroides* and *Parabacteroides* were positively correlated with histological mucosal injury. Changes in *Odoribacter*, *Blautia*, *Oscillibacter*, and *Ruminococcaceae* were negatively correlated with body weights, whereas positively correlated with serum IL-6. *Ruminiclostridium_6*, *Ruminococcaceae_UCG-014*, *Alistipes*, *Lachnospiraceae*, and *Lachnospiraceae_NK4A136_group* were all positively correlated with serum IL-6. *Prevotellaceae_UCG-001* and *Akkermansia* were positively correlated with serum TNF-α. These results further support the involvement of *Prevotellaceae* in the colitis in Rgmb-deficient mice.

### *Prevotellaceae* Was Depleted at the Early Inflammatory Stage of Colitis Associated Colon Cancer in Rgmb-Deficient Mice

**FIGURE 4:**
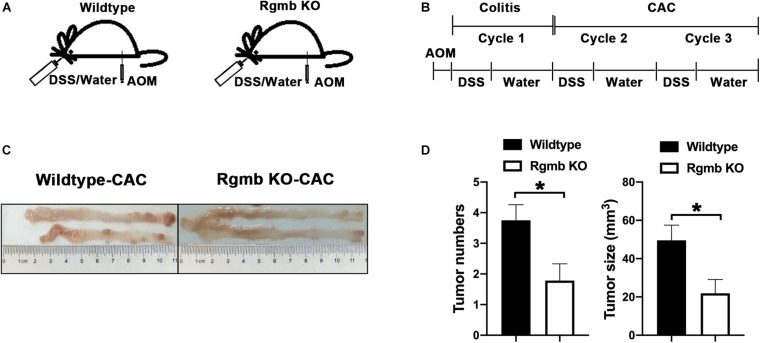
Reduced CAC formation in Rgmb-deficient mice. **(A,B)** The schematic diagrams showing the protocol for induction of CAC in heterozygous global Rgmb-knockout (Rgmb^±^) and wildtype (Rgmb^+/+^) mice. Cycle 1 represents the inflammatory stage before CAC formation. Cycle 2 represents the CAC formation stage following inflammatory conditions. **(C)** Macroscopic images of the AOM/DSS-induced tumors in Rgmb-knockout mice and wildtype littermates. **(D)** The histograms of tumor number and size of CAC tissues. *n* = 9 and *P < 0.05.

Since IBD is a precancerous condition, and colitis has a close relationship with colon cancer, we, therefore, induced colitis associated CRC (CAC) in Rgmb global knockout (Rgmb^*gKO*^) and wildtype (Rgmb^+/+^) mice (Figure 4A and Supplementary Figure 3a). Since homozygous Rgmb knockout mice die within 2–3 weeks after birth (Xia et al., 2011), we used heterozygous Rgmb knockout mice. We analyzed heterozygous Rgmb knockout mice under basal conditions and observed depletion of *Prevotellaceae* and decreased ratio of *Prevotellaceae* to *Muribaculaceae* in heterozygous Rgmb knockout mice compared with wildtype mice (Supplementary Figures S3b,c). Analysis of similarities (ANOSIM) of the bacterial community composition showed that there was no difference in the baseline microbial community composition between global and inducible Rgmb knockout mice (Supplementary Figure 3d; *R* = −0.035, *P* = 0.496).

**FIGURE 5:**
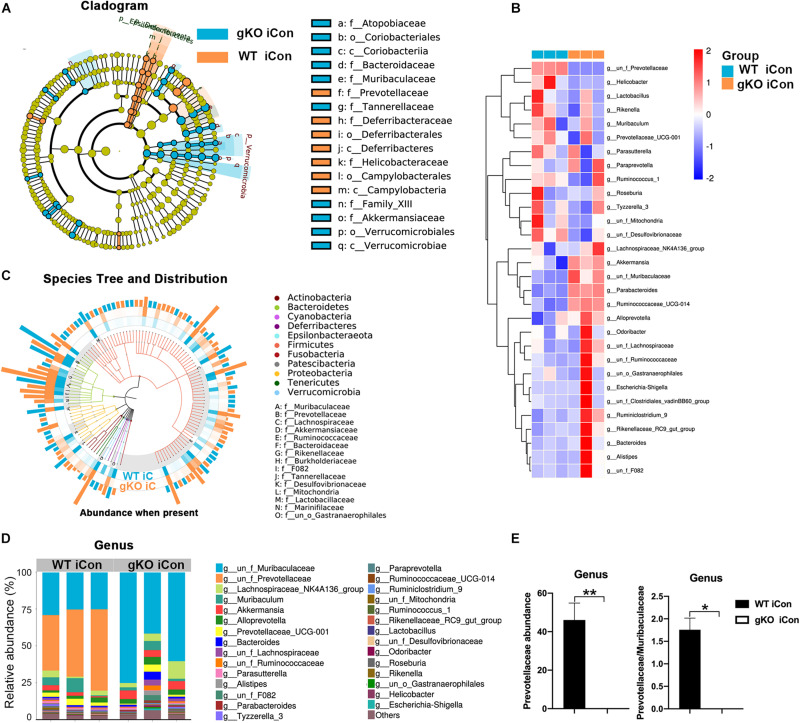
Dysbiosis of gut microbiota in global Rgmb knockout mice at the inflammatory stage of CAC. **(A)** The cladogram of evolution relationship between gut microbiota from heterozygous global Rgmb knockout (Rgmb^±^) and wildtype (Rgmb^+/+^) mice at the inflammatory stage of CAC. **(B)** Heatmap on the relative abundance of gut microbiota from global Rgmb knockout and control mice at the inflammatory stage of CAC. **(C)** The species tree and distribution of gut bacterium at species level. **(D)** The relative abundance of gut microbiota at genus level. **(E)** The relative abundance of *Prevotellaceae* (left panel) and the ratio of *Prevotellaceae* to *Muribaculaceae* (right panel) at genus level. *P < 0.05 and **P < 0.01.

We then induced CAC by three cycles of DSS following intraperitoneal injection of AOM (Figure 4B). Based on the effects of Rgmb deficiency on the gut microbiota in the DSS model, we expected to see increased CAC formation in Rgmb KO mice. To our surprise, Rgmb knockout mice showed decreases in tumor number and size (Figures 4C,D) compared to wildtype mice. We, therefore, asked whether the gut microbiota were differentially regulated by RGMb in the CAC model than in DSS model. To test this notion, we analyzed the gut microbiota at different time points of the CAC induction. We first collected fresh feces for 16S rRNA sequencing after completion of the first cycle of DSS treatment, a stage when the mice were in the inflammatory condition. Observed OTUs indicated significant compositional discriminations of fecal microbiota between Rgmb KO and wildtype colitic mice. Several bacterial OTUs were differentially abundant in Rgmb-deficient mice compared with wildtype ones in different taxonomic ranks (Figures 5A,B). Moreover, bacterial richness was also altered in Rgmb-deficient mice. Enrichments of *Muribaculaceae*, *Akkermansiaceae*, *Bacteroidaceae*, *Rikenellaceae*, F082, *Tannerellaceae*, and *Marinifilaceae* and *Gastranaerophilales*, and reduction of *Prevotellaceae*, *Lachnospiraceae*, *Ruminococcaceae*, *Burkholderiaceae*, *Desulfovibrionaceae*, *Mitochondria*, and *Lactobacillaceae* were found in Rgmb-deficient mice (Figure 5C). Importantly, the depletion of *Prevotellaceae* and the decreased ratio of *Prevotellaceae* to *Muribaculaceae* were observed in Rgmb-deficient mice (Figures 5D,E). These results suggest that there are similar alterations in Rgmb-deficient mice between DSS treatment and the early stage of CAC.

We then analyzed the gut microbiota after completion of the three cycles of DSS treatments when CAC are at advanced stages. The abundance of *Prevotellaceae* and the ratio of *Prevotellaceae* to *Muribaculaceae* were no longer different between Rgmb knockout and wildtype CAC mice (Supplementary Figures 3e,f). These results suggest that Rgmb-deficiency leads to *Prevotellaceae* deletion and gut microbiota dysbiosis during the early stage but not at the late stage of CAC.

## Discussion

Inflammatory bowel disease including UC and CD manifests as relapsing and remitting mucosal inflammation, which may result in progressive bowel damage. IBD symptoms such as strictures, fistula, and abscesses have substantial impacts on a patient’s quality of life. In the most severe cases, IBD may cause morbidity (Feuerstein et al., 2020; Torres et al., 2020). However, the precise etiology of IBD remains incompletely understood. Accumulating evidence demonstrated that dysbiosis of fecal and mucosa-associated microbiota plays a role in the occurrence and development of IBD (Chen et al., 2014; Franzosa et al., 2019). IBD presents altered gut microbiome and immune abnormalities (Schirmer et al., 2019). Rebuilding the normal gut microbiota has become a new strategy for the IBD treatment (Yilmaz et al., 2019; Sokol et al., 2020).

As a BMP co-receptor, RGMb plays an important role in the intestinal tract. RGMb promoted CRC via Erk1/2-BMP4-Smad1/5/8 pathways and induced oxaliplatin resistance by inhibited JNK and p38 MAPK activation (Shi et al., 2015, 2016). Previous studies showed that RGMb is highly expressed in RAW264.7 and J774 macrophage cell lines to negatively regulate IL-6 expression in a BMP ligand-dependent manner. Furthermore, IL-6 is upregulated in macrophages and dendritic cells derived from whole lung tissue of Rgmb knockout mice compared to respective cells derived from wildtype littermates. These results indicate that RGMb is an important negative regulator of IL-6 expression in immune cells, and that Rgmb-deficient mice may be a useful model for exploring immune and inflammatory disorders in colon (Xia et al., 2011). Our present study showed that RGMb expression in the colon was increased by DSS stimulation. Rgmb deletion aggravated DSS-induced colitis. These results are consistent with our previous findings, and further support that RGMb exerts anti-inflammatory effects.

Programmed death ligand 2 is a ligand of PD-1. It has been reported that PD-L2 and BMP-2/4 bind to distinct sites on RGMb (Xiao et al., 2014). RGMb is highly expressed in normal resting lung interstitial macrophages and alveolar epithelial cells, whereas PD-L2 is expressed in lung dendritic cells (Larsen et al., 2019). Blockade of the interaction between RGMb and PD-L2 markedly interfered with the initial T cell expansion and impaired the development of respiratory tolerance. PD-1 inhibits antitumor immunity of T cells, while RGMb regulates respiratory immunity (Xiao et al., 2014; Ohaegbulam et al., 2015). Therefore, RGMb may be an important player in the PD-1 complexes. It would be very interesting to study whether the interaction of RGMb and PD-L2 plays a role in the altered gut microbiota and colitis.

In the inducible Rgmb KO mice, there were significant alterations in both α-diversity and β-diversity, exhaustion of *Prevotellaceae*, and dominance of *Muribaculaceae* in fecal microbiota. Importantly, these phenotypes observed in Rgmb^*igKO*^ were also found in our global Rgmb knockout (Rgmb^*gKO*^) mice. These results collectively suggest that Rgmb deficiency leads to dysbiosis of gut microbiota under basal conditions.

Rgmb^*igKO*^ showed a considerable variation of species and community richness in colitis induced by DSS. The shifted dominance of bacteria observed under basal conditions persisted in Rgmb^*igKO*^ mice even after induction of colitis, which was associated with more severe colitis in Rgmb^*igKO*^ mice. *Prevotellaceae* was almost exhausted at species, family, and genus levels in Rgmb^*igko*^ mice. Three enterotypes (robust clusters) of the human gut microbiome have been identified by the different levels of one of three genera: *Bacteroides* (Enterotype 1), *Prevotella* (Enterotype 2), and *Ruminococcus* (Enterotype 3), which are mostly driven by species composition (Arumugam et al., 2011; Gorvitovskaia et al., 2016; Levy et al., 2020). Here, we demonstrated Rgmb-deficiency shifted the dominance from *Prevotellaceae to Muribaculaceae*, thus providing a good example of host and microbial communications, which may be useful to manipulate the host-microbial symbiotic states.

*Prevotellaceae* exhibited a negative correlation with inflammation in Rgmb-deficient mice with colitis. Furthermore, *Prevotellaceae* dominance even existed in Rgmb-deficient mice under basal conditions. Therefore, *Prevotellaceae* is very likely to be the cause instead of the consequence of the heightened colitis in Rgmb-deficient mice. Nevertheless, whether the shifted dominance of bacteria plays a role in the elevated colitis in Rgmb knockout mice remains to be further clarified.

Colitis has a close relationship with colon cancer; thus, we studied the effects of Rgmb deletion on CAC development using the AOM/DSS model. Rgmb-deficient mice had dramatic alterations in gut microbiota compared to wildtype mice during the early stage of CAC, and the alterations were similar to those observed in the DSS model. However, after the completion of the three cycles of when CAC are already formed, *Prevotellaceae* abundance was no longer different between Rgmb KO and wildtype mice. Therefore, RGMb may play a stage-dependent role in the gut microbiota during CAC development. Even more strikingly, the tumor number and size were reduced in Rgmb KO mice compared with wildtype mice. These results are paradoxical but suggest that some yet-to-be-determined autonomous effects of RGMb on colonic epithelial cells may have overridden the effects of RGMb on the gut microbiota. The molecular mechanisms of RGMb’s stimulatory effects on CAC are currently under active investigation in the laboratory.

Taken together, our results showed that Rgmb deletion led to gut microbiota dysbiosis and depletion of *Prevotellaceae*, and these changes may account for the elevated colitis.

## Data Availability Statement

The original contributions presented in the study are publicly available. These data can be found here: NCBI SRA and accession PRJNA690936, https://www.ncbi.nlm.nih.gov/bioproject/PRJNA690936.

## Ethics Statement

The animal study was reviewed and approved by the Ethics Committee for Animal Research of Jinan University, Guangzhou, China.

## Author Contributions

YS, YX, and ST conceived and designed the experiments. YX provided RGMb knockout mice. YS, LZ, and YL harvested mice and performed animal experiments. LZ, YL, and MW collected the fecal samples. YC and SF extracted the DNA. YS, YX, and ST analyzed the sequencing data and wrote the manuscript. All authors read and approved the final version of the manuscript.

## Conflict of Interest

The authors declare that the research was conducted in the absence of any commercial or financial relationships that could be construed as a potential conflict of interest.

## Supplementary Material

Supplementary Figure 1 Rgmb-deficiency induced dysbiosis of gut microbiota in mice. **(a)** The schematic diagram represented inducible Rgmb knockout mice model. **(b)** The cladogram of evolution relationship between gut microbiota from Rgmb-deficient and control mice. **(c)** Heatmap represented the relative abundance of gut microbiota from Rgmb-deficient and control mice at genus level. **(d)** The species tree and distribution of gut bacterium at species level in Rgmb-deficient and control mice. **(e)** The relative abundance of gut microbiota in Rgmb-deficient and control mice at genus level. **(f)** The relative abundance of *Prevotellaceae* (left panel) and the ratio of *Prevotellaceae* to *Muribaculaceae* (right panel) in Rgmb-deficient and control mice at genus level. ^∗^*P* < 0.05, ^∗∗^*P* < 0.01.

Supplementary Figure 2 The relative abundance of gut microbiota in Rgmb-deficient and control mice at genus level. ^∗^*P* < 0.05, n.s., not significant.

Supplementary Figure 3 The relative abundance of gut microbiota in Rgmb-deficient and wildtype CAC mice at genus level. **(a)** The schematic diagram represented Rgmb-knockout (Rgmb^+/–^) and wildtype (Rgmb^+/+^) mice model. **(b)** Heatmap represented the relative abundance of gut microbiota from Rgmb-deficient and wildtype mice at genus level. **(c)** The relative abundance of *Prevotellaceae* and the ratio of *Prevotellaceae* to *Muribaculaceae* in Rgmb-deficient and control mice at genus level. **(d)** Analysis of similarities (ANOSIM) between global Rgmb knockout and inducible Rgmb knockout mice. R = −0.035, *P* = 0.496. **(e)** Heatmap represented the relative abundance of gut microbiota from Rgmb-deficient and wildtype CAC mice at genus level. **(f)** The relative abundance of *Prevotellaceae* and the ratio of *Prevotellaceae* to *Muribaculaceae* in Rgmb-deficient and control CAC mice at genus level. ^∗∗^*P* < 0.01, n.s. not significant.

Supplementary Table 1 Correlation statistics between gut microbiota and inflammation of Rgmb-deficient mice with colitis.
