# Supplementary figures and images for: Repulsive Guidance Molecule b Deficiency Induces Gut Microbiota Dysbiosis and Increases the Susceptibility to Intestinal Inflammation in Mice
Source: Front Microbiol. 2021 Apr 28;12:648915. doi: 10.3389/fmicb.2021.648915 (PMC8113641; doi:10.3389/fmicb.2021.648915)

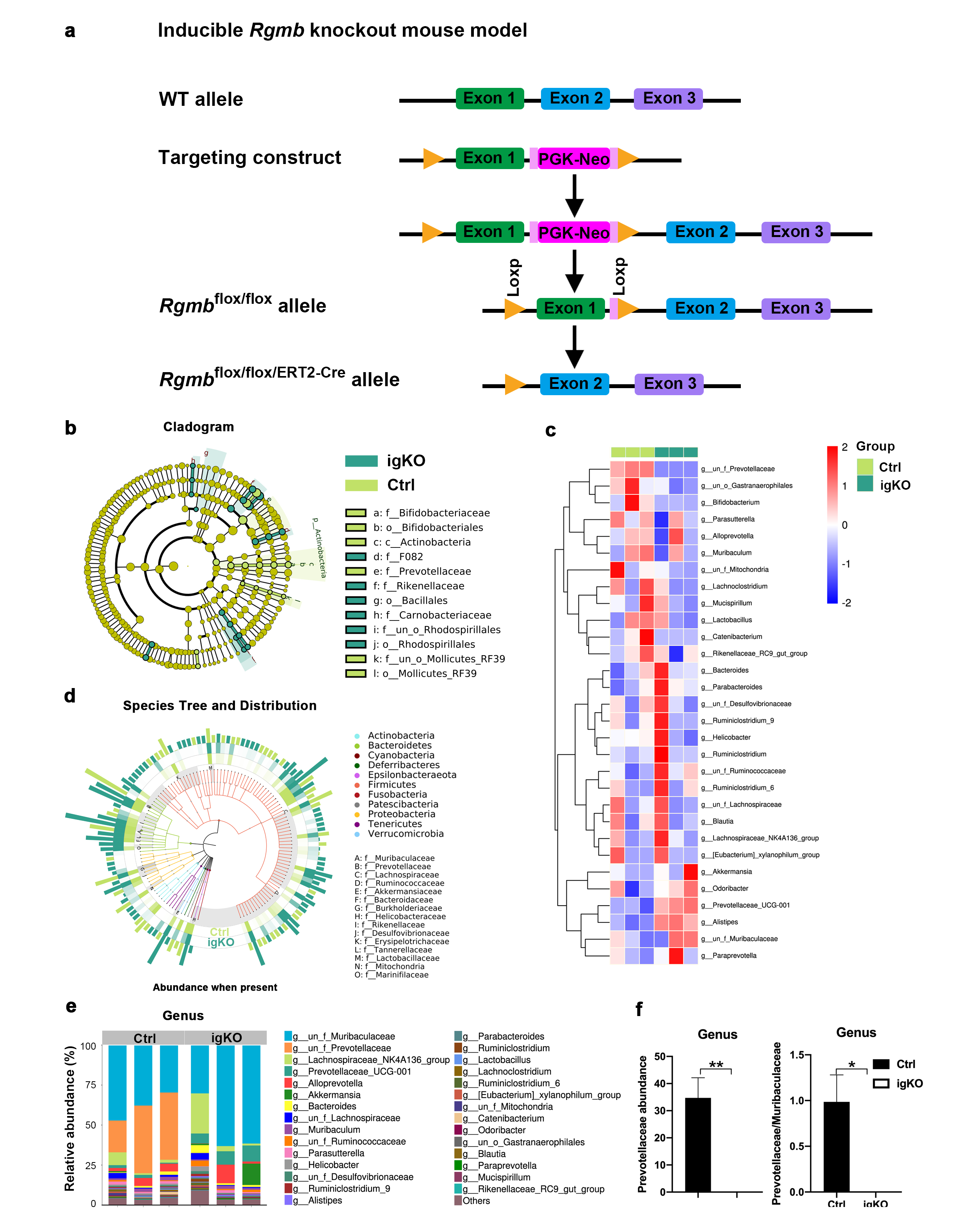

Supplement: Supplementary Figure 1 — Rgmb-deficiency induced dysbiosis of gut microbiota in mice. (a) The schematic diagram represented inducible Rgmb knockout mice model. (b) The cladogram of evolution relationship between gut microbiota from Rgmb-deficient and control mice. (c) Heatmap represented the relative abundance of gut microbiota from Rgmb-deficient and control mice at genus level. (d) The species tree and distribution of gut bacterium at species level in Rgmb-deficient and control mice. (e) The relative abundance of gut microbiota in Rgmb-deficient and control mice at genus level. (f) The relative abundance of Prevotellaceae (left panel) and the ratio of Prevotellaceae to Muribaculaceae (right panel) in Rgmb-deficient and control mice at genus level. ∗P < 0.05, ∗∗P < 0.01. [file Image_1.TIF]

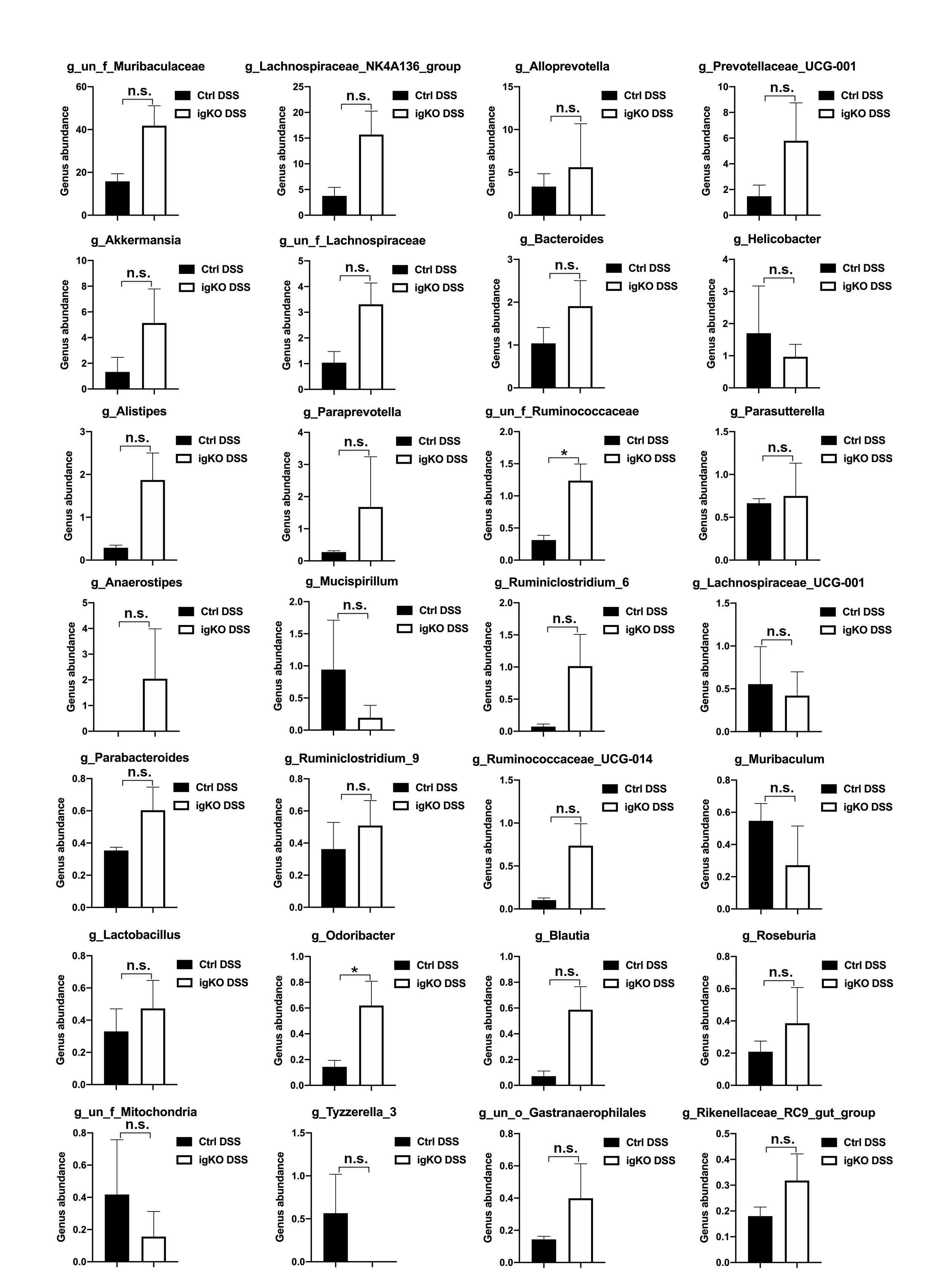

Supplement: Supplementary Figure 2 — The relative abundance of gut microbiota in Rgmb-deficient and control mice at genus level. ∗P < 0.05, n.s., not significant. [file Image_2.TIF]

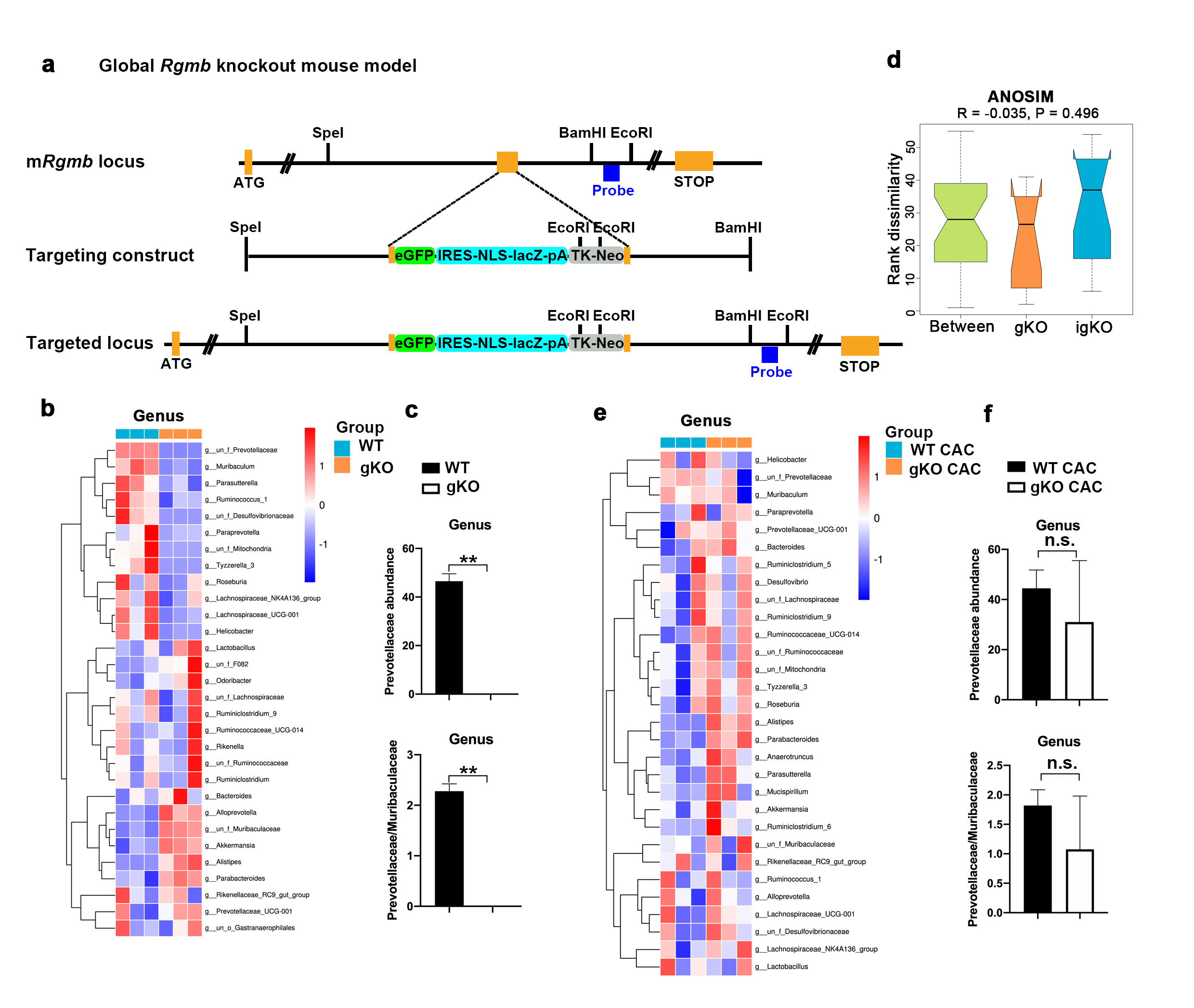

Supplement: Supplementary Figure 3 — The relative abundance of gut microbiota in Rgmb-deficient and wildtype CAC mice at genus level. (a) The schematic diagram represented Rgmb-knockout (Rgmb+/–) and wildtype (Rgmb+/+) mice model. (b) Heatmap represented the relative abundance of gut microbiota from Rgmb-deficient and wildtype mice at genus level. (c) The relative abundance of Prevotellaceae and the ratio of Prevotellaceae to Muribaculaceae in Rgmb-deficient and control mice at genus level. (d) Analysis of similarities (ANOSIM) between global Rgmb knockout and inducible Rgmb knockout mice. R = −0.035, P = 0.496. (e) Heatmap represented the relative abundance of gut microbiota from Rgmb-deficient and wildtype CAC mice at genus level. (f) The relative abundance of Prevotellaceae and the ratio of Prevotellaceae to Muribaculaceae in Rgmb-deficient and control CAC mice at genus level. ∗∗P < 0.01, n.s. not significant. [file Image_3.TIF]
