# Supplementary material for: Repulsive Guidance Molecule b Deficiency Induces Gut Microbiota Dysbiosis and Increases the Susceptibility to Intestinal Inflammation in Mice
Source: Front Microbiol. 2021 Apr 28;12:648915. doi: 10.3389/fmicb.2021.648915 (PMC8113641; doi:10.3389/fmicb.2021.648915)
Supplement: Supplementary Table 1 — Correlation statistics between gut microbiota and inflammation of Rgmb-deficient mice with colitis. [file Table_1.pdf]

Supplementary Table 1. Correlation statistics between gut microbiota and inflammation of Rgmb-deficient mice with colitis.

No.	Bacteria	Histology		DAI		Weight		IL-6		IL-10		TNF-α		IFN-γ	
		r	*P*	*r*	*P*	*r*	*P*	*r*	*P*	*r*	*P*	*r*	*P*	*r*	*P*
1	*Prevotellaceae*	-0.90067	0.014	-0.74904	0.087	0.987203	0.000	-0.9266	0.008	-0.88482	0.019	-0.87395	0.023	-0.94137	0.005
2	*Muribaculum*	-0.28359	0.586	-0.10729	0.840	0.601006	0.207	-0.45159	0.369	-0.34227	0.507	-0.65773	0.156	-0.35896	0.485
3	*Roseburia*	0.074895	0.888	0.143795	0.786	-0.44910	0.372	0.531535	0.278	0.064143	0.904	0.488377	0.326	0.128731	0.808
4	*Gastranaerophilales*	0.139217	0.793	0.417882	0.410	-0.58620	0.221	0.668337	0.147	0.303788	0.558	0.694279	0.126	0.366284	0.475
5	*Anaerostipes*	0.141653	0.789	0.421354	0.405	-0.50523	0.307	0.714663	0.111	0.135269	0.798	0.500858	0.312	0.278619	0.593
6	*Rikenellaceae_RC9_gut_group*	0.354479	0.491	0.358761	0.485	-0.57958	0.228	0.755072	0.083	0.152259	0.773	0.442991	0.379	0.328919	0.524
7	*Odoribacter*	0.525220	0.285	0.520307	0.290	-0.82795	0.042	0.868329	0.025	0.512729	0.298	0.793623	0.059	0.592408	0.215
8	*Blautia*	0.591063	0.217	0.621566	0.188	-0.84633	0.034	0.93673	0.006	0.534745	0.274	0.760184	0.079	0.659901	0.154
9	*Oscillibacter*	0.610274	0.198	0.607479	0.201	-0.87548	0.022	0.92721	0.008	0.576004	0.232	0.804461	0.054	0.685495	0.133
10	*Ruminiclostridium_6*	0.467306	0.350	0.594374	0.213	-0.69204	0.128	0.890306	0.017	0.351826	0.494	0.575730	0.232	0.511207	0.300
11	*Alistipes*	0.511717	0.299	0.623455	0.186	-0.80374	0.054	0.919448	0.009	0.501721	0.311	0.756806	0.082	0.605103	0.203
12	*Ruminococcaceae_UCG-014*	0.521981	0.288	0.651955	0.161	-0.77944	0.068	0.930604	0.007	0.482472	0.332	0.707583	0.116	0.597234	0.211
13	*Parasutterella*	0.095722	0.857	-0.10285	0.846	-0.21069	0.689	-0.14061	0.790	0.373907	0.465	0.389867	0.445	0.232110	0.658
14	*Prevotellaceae_UCG-001*	0.182667	0.729	0.543513	0.265	-0.64477	0.167	0.647636	0.164	0.51133	0.300	0.825706	0.043	0.534710	0.274
15	*Akkermansia*	0.198436	0.706	0.545545	0.263	-0.61454	0.194	0.524068	0.286	0.628774	0.181	0.842841	0.035	0.598989	0.209
16	*Tyzzerella_3*	-0.19670	0.709	-0.73387	0.097	0.422329	0.404	-0.50848	0.303	-0.61135	0.197	-0.61996	0.189	-0.50246	0.310
17	*Mucispirillum*	-0.03809	0.943	-0.64182	0.169	0.351209	0.495	-0.4264	0.399	-0.52243	0.288	-0.61738	0.192	-0.39670	0.436
18	*Mitochondria*	0.093422	0.86	-0.54852	0.260	0.280131	0.591	-0.34544	0.502	-0.43073	0.394	-0.59117	0.217	-0.30076	0.562
19	*Ruminiclostridium_9*	0.324269	0.531	-0.01829	0.973	-0.41010	0.419	0.475477	0.341	-0.06104	0.909	0.186012	0.724	0.132536	0.802
20	*Helicobacter*	0.022329	0.967	-0.47618	0.340	0.120379	0.820	-0.14174	0.789	-0.43928	0.383	-0.37462	0.464	-0.25071	0.632
21	*Lachnospiraceae_UCG-001*	-0.02050	0.969	-0.39368	0.440	-0.01257	0.981	0.017437	0.974	-0.39177	0.442	-0.16765	0.751	-0.20914	0.691

22	*Bacteroides*	0.813351	0.049	0.184322	0.727	-0.46319	0.355	0.392237	0.442	0.402802	0.428	0.146077	0.782	0.41862	0.409
23	*Lactobacillus*	0.594868	0.213	-0.04099	0.939	-0.25864	0.621	0.277158	0.595	0.062065	0.907	-0.08866	0.867	0.113688	0.830
24	*Alloprevotella*	0.472541	0.344	0.127455	0.810	-0.07773	0.884	0.100097	0.850	0.182918	0.729	-0.15526	0.769	0.151398	0.775
25	*Paraprevotella*	0.621909	0.187	0.391639	0.443	-0.27925	0.592	0.326323	0.528	0.382837	0.454	0.03308	0.95	0.41606	0.412
26	*Ruminococcaceae*	0.768707	0.074	0.66883	0.146	-0.84168	0.036	0.943538	0.005	0.593227	0.215	0.654798	0.158	0.713169	0.112
27	*Lachnospiraceae_NK4A136_group*	0.749628	0.086	0.574179	0.233	-0.74386	0.09	0.868648	0.025	0.472773	0.344	0.498418	0.314	0.615697	0.193
28	*Lachnospiraceae*	0.67052	0.145	0.496199	0.317	-0.78980	0.062	0.885129	0.019	0.436871	0.386	0.600404	0.208	0.571046	0.237
29	*Muribaculaceae*	0.696423	0.124	0.652117	0.160	-0.79884	0.057	0.584538	0.223	0.955942	0.003	0.857554	0.029	0.898544	0.015
30	*Parabacteroides*	0.826381	0.043	0.471811	0.345	-0.56016	0.248	0.424971	0.401	0.735909	0.095	0.3998	0.432	0.67487	0.141
